# Murine Models of Steroid Refractory Graft-versus-Host Disease

**DOI:** 10.1038/s41598-018-30814-x

**Published:** 2018-08-20

**Authors:** Tomomi Toubai, Corinne Rossi, Isao Tawara, Chen Liu, Cynthia Zajac, Katherine Oravecz-Wilson, Daniel Peltier, Yaping Sun, Hideaki Fujiwara, Shin-Rong Wu, Mary Riwes, Israel Henig, Stephanie Kim, Pavan Reddy

**Affiliations:** 1 Department of Internal Medicine, Division of Hematology and Oncology, University of Michigan Comprehensive Cancer Center, Ann Arbor, MI USA; 2 Department of Internal Medicine III, Division of Hematology and Cell Therapy, Yamagata University Faculty of Medicine, Yamagata, Japan; 3 Present Address: Department of Pediatrics, Division of Hematology and Oncology, University Hospital of Heidelberg, Heidelberg, Germany; 4 Department of Hematology and Oncology, Mie University Graduate School of Medicine, Mie, Japan; 5 Department of Pathology and Laboratory Medicine, Rutgers-Robert Wood Johnson Medical School, New Brunswick, NJ USA; 6 Division of Hematology and Oncology, Department of Pediatrics, University of Michigan, Ann Arbor, MI USA

## Abstract

Corticosteroids are the first line therapy for acute graft-versus-host disease (GVHD). However, the outcome of steroid refractory GVHD (SR-GVHD) is poor due to a lack of effective treatments. The development of therapies for SR-GVHD is limited by an incomplete understanding of its pathophysiology partly because of the absence of clinically relevant animal models of SR-GVHD. Here we addressed the need for a SR-GVHD animal model by developing both MHC matched multiple minor histocompatibility antigens (miHAs) mismatched and MHC mismatched haploidentical murine models of SR-GVHD. We demonstrate that animals can develop SR-GVHD regardless of whether steroids are initiated early or late post allogeneic bone marrow transplantation (allo-BMT). In general, we observed increased GVHD specific histopathological damage of target organs in SR-GVHD animals relative to steroid responsive animals. Interestingly, we found no significant differences in donor T cell characteristics between steroid refractory and responsive animals suggesting that donor T cell independent mechanisms may play more prominent roles in the pathogenesis of SR-GVHD than was considered previously.

## Introduction

Acute graft-versus-host disease (GVHD) is a major complication of allogeneic cell transplantation (allo-HCT). For decades, steroids have remained the first-line treatment for acute GVHD; however, 30–50% of patients develop steroid refractory GVHD (SR-GVHD), which portends an extremely poor prognosis^1^. Many agents^2^ have shown encouraging phase II response rates, but none have demonstrated a long term survival advantage including three tested in randomized controlled trials^3^. The lack of effective SR-GVHD treatments is likely compounded by our incomplete understanding of its pathophysiology. There are many reasons why SR-GVHD pathogenesis is poorly understood, but one reason may be the lack of clinically relevant animal models. Herein we generated murine SR-GVHD models, which surprisingly suggest that donor T cell independent mechanisms may play a more important role in SR-GVHD pathogenesis than previously appreciated.

## Results and Discussion

### Dexamethasone does not uniformly mitighate GVHD severity or survival

**Figure 1:**
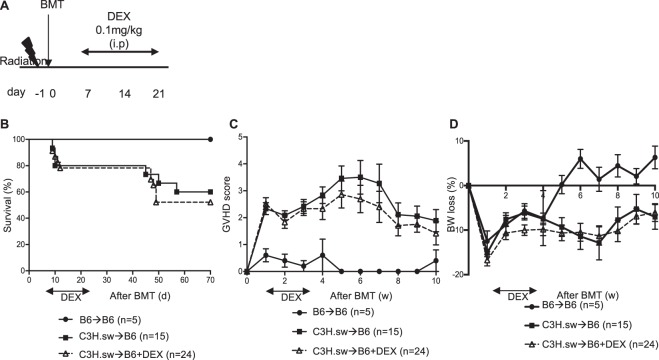
Dexamethasone treatment in murine BMT model. **(A–D)** B6 WT animals received 10 Gy on day −1 and were transplanted with 3.0 × 10^6^ CD90.2^+^ splenic T cells along with 5 × 10^6^ T cell depleted (TCD)-bone marrow (BM) from either syngeneic B6 or allogeneic MHC matched multiple minor antigens (miHAs) mismatched C3H.sw donors. Recipient animals were treated intraperitoneally with 0.1 mg/kg/day of dexamethasone (DEX) from day +7 thru +21 after allo-BMT. **(A)** Experimental schema. **(B)** Survival. **(C)** GVHD clinical score. **(D)** The percentage of body weight (BW) loss. n = 5–24 per group. Data shown are pooled from four independent experiments. Error bars show the mean ± SEM.

Because most clinical transplants are human leukocyte antigen (HLA) matched, we first utilized the major histocompatibility (MHC) matched multiple minor histocompatibility antigens (miHAs) mismatched C3H.sw→B6 BMT model. Recipient B6 WT animals were lethally irradiated and transplanted with splenic T cells along with bone marrow (BM) cells from either syngeneic B6 or allogeneic C3H.sw donors. Dexamethasone (DEX) was delivered from day +7 to day +21 at a dose of 0.1 mg/kg (Fig. 1A) which was intentionally lower than in previous studies^4,5^ so as to better reflect the prednisolone-equivalent dose used clinically. Surprisingly, despite dexamethasone treatment, there were no significant differences in overall survival (OS), GVHD clinical scores, or the percentages of body weight (BW) lost between treated and untreated control groups in our model (Fig. 1B–D). To determine whether higher doses of DEX might improve GVHD response rate in this model, we also tested high dose DEX (10 mg/kg)^4^. In data not shown, even the higher dose did not demonstrate any significant differences in OS or GVHD severity in our model system.

### Dexamethasone-treated animals can be divided into three groups based on response in multiple BMT models

**Figure 2:**
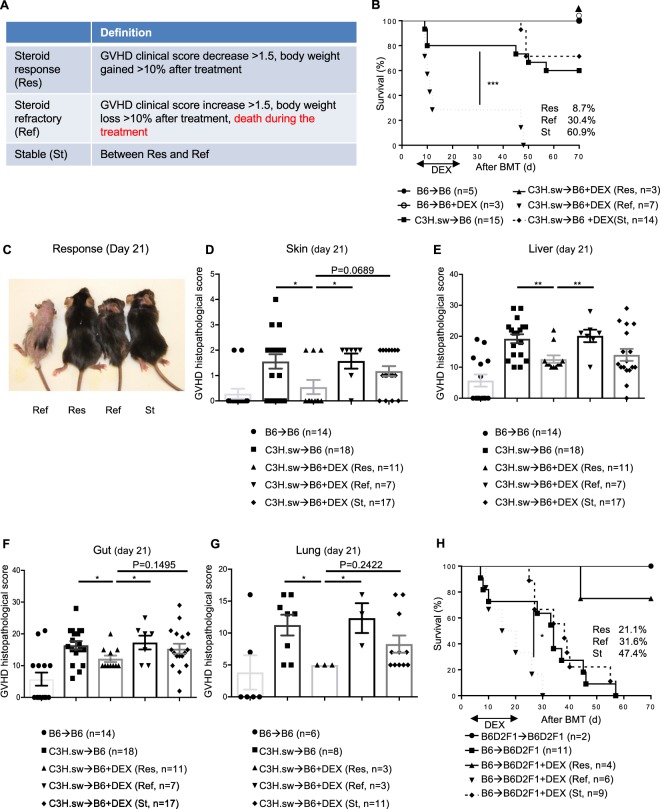
Early DEX treatment SR-GVHD murine BMT models. **(A)** Our criteria of steroid responsiveness in murine BMT models. **(B–G)** B6 WT animals received 10 Gy on day −1 and were transplanted with 3.0 × 10^6^ CD90.2^+^ splenic T cells along with 5 × 10^6^ TCD-BM from either syngeneic B6 or allogeneic MHC matched multiple miHAs mismatched C3H.sw donors. Recipient animals were treated intraperitoneally with 0.1 mg/kg/day of dexamethasone (DEX) from day +7 thru +21 after allo-BMT. **(B)** Survival. The allogeneic DEX treated animals shown in Fig. 1B were divided into three groups based on our criteria, such as steroid responsive (Res), stable (St) or steroid refractory (Ref) GVHD. n = 3–25 per group. Data shown are pooled from four independent experiments. ***p < 0.001, when Ref animals were compared to other groups. **(C)** Representative picture of skin response to DEX. **(D–G)** Histopathological GVHD score in skin **(D)**, liver **(E)**, gastrointestinal (GI) tract **(F)**, and lung **(G)** on day 14 of DEX treatment (day 21 after allo-BMT) from the different groups in B (n = 3–18 per group). Data are pooled from five independent experiments. *p < 0.05, **p < 0.01. **(H)** B6D2F1 animals received 11 Gy on day −1 and were transplanted with 3.0 × 10^6^ CD90.2^+^ splenic T cells along with 5 × 10^6^ TCD-BM from either syngeneic B6D2F1 or allogeneic MHC mismatched haploidentical B6 donors. Recipient animals were treated intraperitoneally with 0.1 mg/kg/day of DEX from day +7 thru +21 after allo-BMT. Survival is shown (n = 2–11 per group). Data are pooled from two independent experiments. *p < 0.05, when Ref animals were compared to other groups. Error bars show the mean ± SEM.

Because we couldn’t see any therapeutic responses in DEX treated animals, we speculated that treated animals have different responses to DEX in spite of a uniform genetic background and housing conditions. Interestingly, we observed three phenotypes in response to DEX: 1) progressive GVHD characterized by severe peak GVHD clinical scores, progressive BW loss and early mortality; 2) stable GVHD characterized by moderate GVHD clinical scores, moderate BW loss, and minimal late mortality; and 3) steroid-responsive GVHD characterized by near complete normalization of GVHD clinical scores, near complete normalization of BW loss, and no mortality (Fig. 2A). We were able to differentiate among these three phenotypes based on GVHD clinical score and BW on day +21 after allo-BMT which included 14 days of DEX treatment. For the remainder of this article, we refer to the aforementioned three groups as steroid refractory (Ref), stable (St), and responsive (Res) GVHD, respectively. These criteria clearly separated steroid treatment animals into three groups based on mortality (Fig. 2A). Indeed, the survival percentages at the end of the experiments were 100, 73, and 0% for animals with Res, St, and Ref at day 21, respectively (Fig. 2A). Ref animals demonstrated significantly higher day +21 GVHD severity, percent of BW loss, and histopathological scores of GVHD target organs in relative to Res animals (Fig. 2B–G, Supplemental Fig. 1a,b).

To eliminate strain dependent factors, we also examined whether the differential steroid response predicted by our scoring systems holds in an MHC mismatched haploidentical B6→B6D2F1 model. We found a similar differential response to corticosteroids; albeit with a slightly different distribution among the groups and greater mortality in the St and Res groups (Fig. 2H).

To test whether our scoring system specifically separates DEX treated animals relative to control untreated animals, we applied our scoring system to 15 untreated control animals shown in Fig. 2B. No control animals met criteria for DEX-responsive. However, the control animals categorized as DEX refractory demonstrated higher GVHD severity and mortality than those classified as St (Supplemental Fig. 2a,b). These data suggest that our scoring system specifically identifies DEX responsive allogeneic animals.

### Delayed dexamethasone treated animals also separate recipients into 3 response-based groups

**Figure 3:**
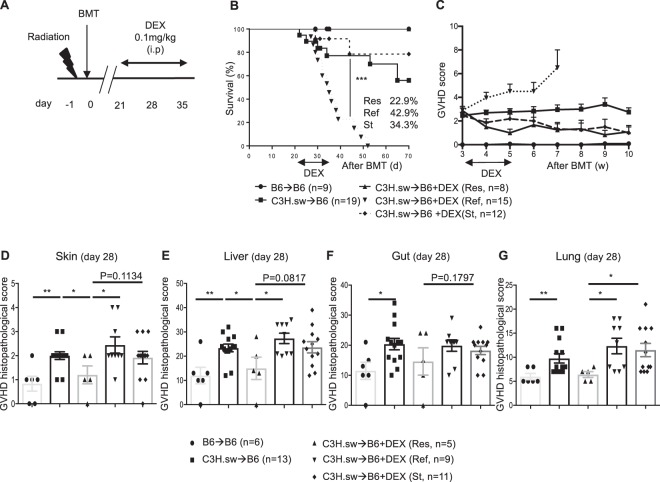
Late DEX treatment SR-GVHD murine BMT model. **(A–G)** B6 WT animals received 10 Gy on day -1 and were transplanted with 3.0 × 10^6^ CD90.2^+^ splenic T cells along with 5 × 10^6^ TCD-BM from either syngeneic B6 or allogeneic MHC matched multiple miHAs mismatched C3H.sw donors. Recipient animals were treated with 0.1 mg/kg/day of DEX from day +21 thru day +35 after allo-BMT. **(A)** The experimental schema. **(B)** Survival and **(C)** GVHD clinical score of A. n = 9–19 per group. DEX treated animals were characterized into three groups as above. ***p < 0.001, when Ref animals were compared to other groups. **(C–G)** Histopathological GVHD score in skin **(D)**, liver **(E)**, GI tract **(F)** and lung **(G)** on day 7 after DEX treatment (day 28 after allo-BMT) from the different group in B and C. n = 5–13 per group. Data are pooled from four independent experiments. *p < 0.05, **p < 0.01. Error bars show the mean ± SEM.

To account for any confounding effect of radiation toxicity, we next sought to determine whether steroid refractoriness can be modelled at a later time-point. To accomplish this, we utilized the same C3H.sw → B6 model except that DEX was administered from day +21, at which point there was already approximately 20% mortality among allogeneic recipients, to day +35 (Fig. 3A). We again obtained similar overall survivals, GVHD clinical scores, and GVHD specific histopathological scores of GVHD target organs as in our early steroid treatment model (Fig. 3B–G). Only GI tract histopathological scores at day 28 after allo-BMT were not significantly different but tended towards decreased GVHD severity than other groups. These data suggest that our scoring system can clearly separate DEX treated animals into Res, Ref, and St subpopulations regardless of whether DEX is delivered early or late post allo-BMT.

### Inflammatory cytokines are negligible role in steroid refractoriness

**Figure 4:**
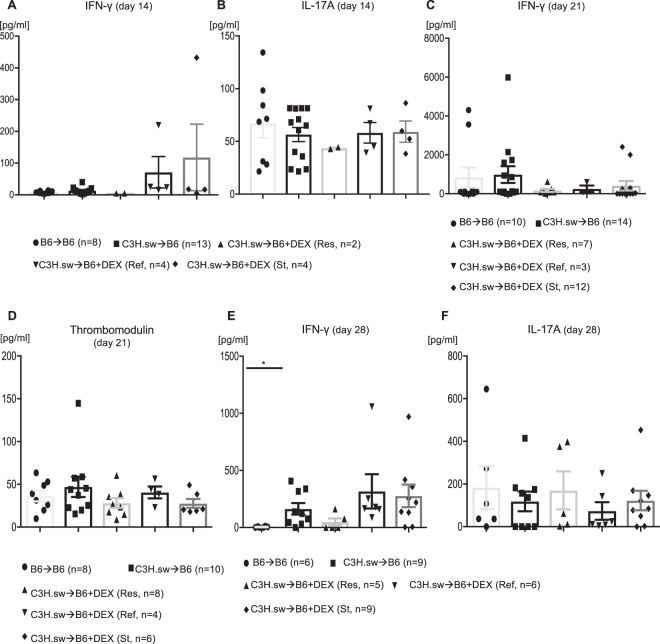
Inflammatory cytokines are negligible for steroid refractoriness. **(A–D)** B6 WT animals received 10 Gy on day −1 and were transplanted with 3.0 × 10^6^ CD90.2^+^ splenic T cells along with 5 × 10^6^ TCD-BM from either syngeneic B6 or allogeneic MHC matched multiple miHAs mismatched C3H.sw donors. Recipient animals were treated intraperitoneally with 0.1 mg/kg/day of dexamethasone (DEX) from day +7 thru +21 after allo-BMT. **(A–B)** Serum IFN-γ **(A)** and IL-17A **(B)** levels 14 days after allo-BMT from the different group in Fig. 2B. n = 2–13. **(C–D)** Serum IFN-γ **(C)** and thrombomodulin **(D)** levels 21 days after allo-BMT from the different group in Fig. 2B. n = 3–14. Data were pooled from five independent experiments. Data were pooled from five independent experiments. **(E–F)** B6 WT animals received 10 Gy on day −1 and were transplanted with 3.0 × 10^6^ CD90.2^+^ splenic T cells along with 5 × 10^6^ TCD-BM from either syngeneic B6 or allogeneic MHC matched multiple miHAs mismatched C3H.sw donors. Recipient animals were treated with 0.1 mg/kg/day of DEX from day +21 thru day +28 after allo-BMT. Serum IFN-γ **(E)** and IL-17A **(F)** levels 14 days after allo-BMT from the different groups in Fig. 3B. n = 5–9. Data were pooled from two independent experiments. Error bars show the mean ± SEM.

Because inflammatory cytokines, such as IFN-γ and IL-17A, exacerbate GVHD severity and mortality^6,7^, we determined whether these cytokines correlated with treatment responses. To our surprise, serum levels of IFN-γ, and IL-17A were not significantly altered in any early or late DEX-treated animals (Fig. 4A–C,E,F). In addition, because clinical steroid refractory GVHD is associated with elevated serum thrombomodulin levels^8^, we assessed this in our model system but couldn’t find any significant difference among DEX treatment groups (Fig. 4D). These data suggest that inflammatory cytokines associated activated inflammatory T cells are dispensable for GVHD response to DEX.

### Donor T cell responses after administration of DEX

**Figure 5:**
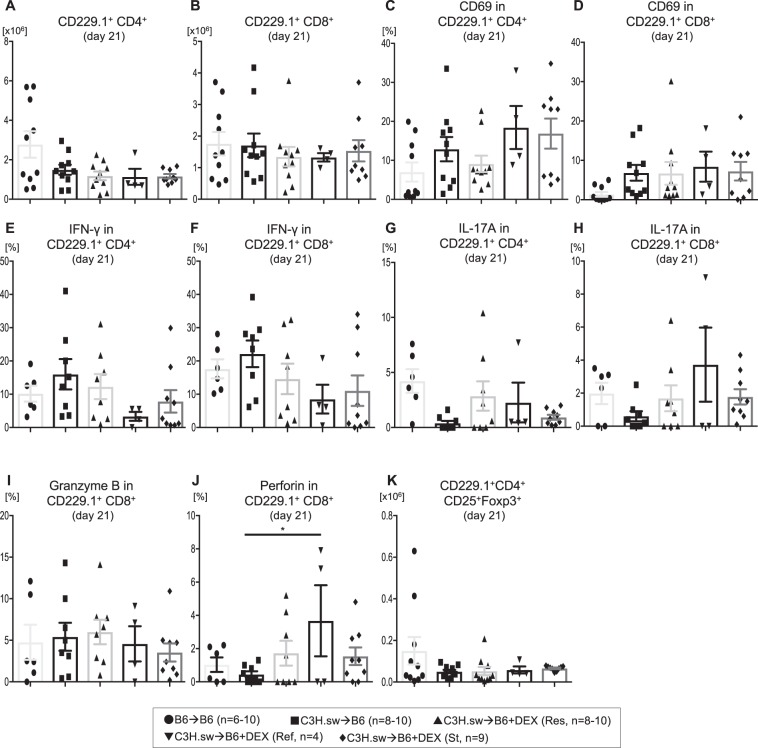
Steroid refractoriness may be mediated by a T cell independent mechanism. **(A–K)** B6 WT animals received 10 Gy on day −1 and were transplanted with 3.0 × 10^6^ CD90.2^+^ splenic T cells along with 5 × 10^6^ TCD-BM from either syngeneic B6 or allogeneic MHC matched multiple miHAs mismatched C3H.sw donors. Recipient animals were treated intraperitoneally with 0.1 mg/kg/day of dexamethasone (DEX) from day +7 thru +21 after allo-BMT. Absolute numbers of donor (CD229.1^+^) CD4^+^ **(A)**, CD8^+^ **(B)** T cells, CD69 expression on CD229.1^+^CD4^+^ **(C)**, CD69 expression on CD229.1^+^CD8^+^ T cells **(D)**, IFN-γ production in CD229.1^+^CD4^+^ **(E)**, IFN-γ production in CD229.1^+^CD8^+^ T cells **(F)**, IL-17A production in CD229.1^+^CD4^+^ **(G)**, L-17A production in CD229.1^+^CD8^+^ **(H)** T cells, granzyme B production in CD229.1^+^CD8^+^ T cells **(I)**, perforin production in CD229.1^+^CD8^+^ T cells and absolute numbers of CD229.1^+^CD4^+^CD25^+^Foxp3^+^ **(J)** T cells in the spleen on day 21 after allo-HCT from the different groups in Fig. 2B is shown. n = 4–10 per group. Data are pooled from five experiments. *p < 0.05. Error bars show the mean ± SEM.

To identify mechanisms critical for steroid refractoriness in our BMT models, we determined whether the refractoriness correlated with persistent proliferation, activation, or enhanced cytokine production in donor T cells from steroid refractory animals. To test this, we isolated splenic T cells from recipient animals and analyzed donor CD229.1^+^CD4^+^ or CD8^+^ T cell expansion, activation (CD69), proinflammatory cytokine production (IFN-γ, IL-17A, granzyme B, perforin), and CD4^+^CD25^+^Foxp3^+^ regulatory T cells (Tregs) by FACS. However, donor CD4^+^ and CD8^+^ T cell expansion in the spleen was similar among these groups at day 21 after allo-BMT (Fig. 5A,B). Furthermore, no significant differences were found in the proportion of activated T cells (Fig. 5C–D). In addition, cytokine production by these T cells was not changed (Fig. 5E-J). We also examined effector (CD44^+^CD62L^−^), memory (CD44^+^CD62L^+^), naïve (CD44^−^CD62L^+^) T cells, or Tregs in the spleen, but these subsets were similar among all allogeneic groups (Fig. 5K, Supplemental Fig. 3).

**Figure 6:**
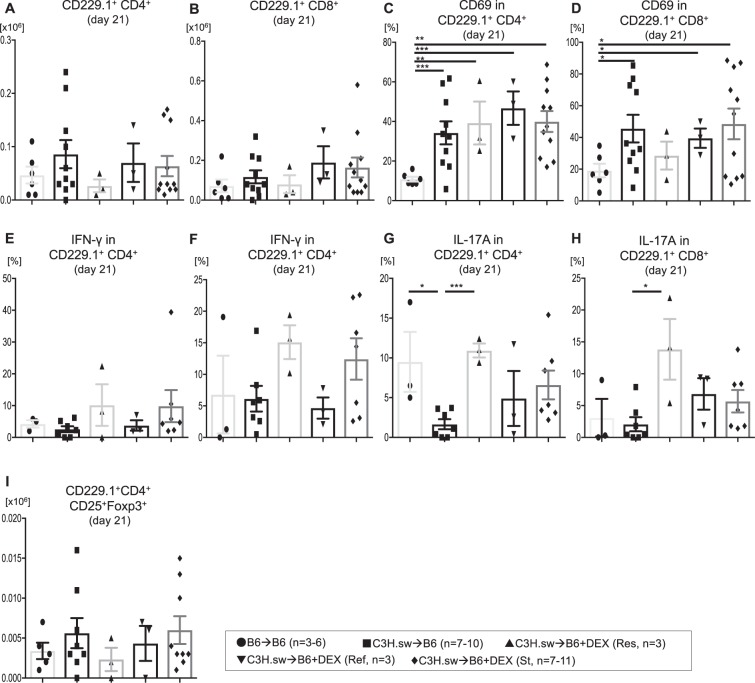
Donor T cells in liver were not involved in steroid refractoriness. **(A–I)** B6 WT animals received 10 Gy on day −1 and were transplanted with 3.0 × 10^6^ CD90.2^+^ splenic T cells along with 5 × 10^6^ TCD-BM from either syngeneic B6 or allogeneic MHC matched multiple miHAs mismatched C3H.sw donors. Recipient animals were treated intraperitoneally with 0.1 mg/kg/day of dexamethasone (DEX) from day +7 thru +21 after allo-BMT. Absolute numbers of donor (CD229.1^+^) CD4^+^ **(A)** and CD8^+^ **(B)** T cells, CD69 expression on CD229.1^+^CD4^+^ T cells **(C)**, CD69 expression on CD229.1^+^CD8^+^ T cells **(D)**, IFN-γ production in CD229.1^+^CD4^+^ T cells **(E)**, IFN-γ production in CD8^+^ T cells **(F)**, IL-17A production in CD229.1^+^CD4^+^ T cells **(G)**, IL-17A production in CD229.1^+^CD8^+^ T cells **(H)**, and absolute numbers of CD229.1^+^CD4^+^CD25^+^Foxp3^+^ **(I)** T cells in the liver on day 21 after allo-HCT from the different groups in Fig. 2B is shown. n = 4–11 per group. Data are pooled from five experiments. ***p < 0.001, **p < 0.01, *p < 0.05. Error bars show the mean ± SEM.

**Figure 7:**
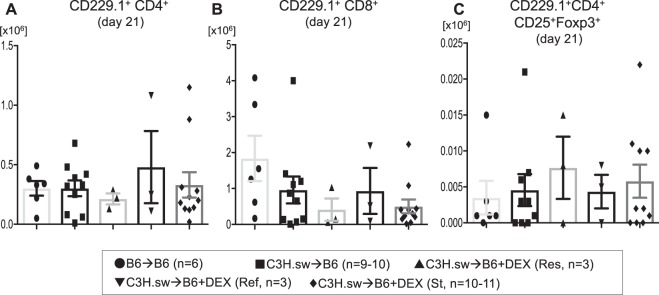
Donor intra epithelial lymphocytes do not differ by steroid responsiveness. **(A–C)** B6 WT animals received 10 Gy on day −1 and were transplanted with 3.0 × 10^6^ CD90.2^+^ splenic T cells along with 5 × 10^6^ TCD-BM from either syngeneic B6 or allogeneic MHC matched multiple miHAs mismatched C3H.sw donors. Recipient animals were treated intraperitoneally with 0.1 mg/kg/day of dexamethasone (DEX) from day +7 thru +21 after allo-BMT. Absolute numbers of donor (CD229.1^+^) CD4^+^ **(A)** T cells, CD8^+^ **(B)** T cells, and absolute numbers of CD229.1^+^CD4^+^CD25^+^Foxp3^+^ **(C)** T cells in the intestine on day 21 after allo-HCT from the different the groups in Fig. 2B is shown. n = 4–11 per group. Data are pooled from five experiments. Error bars show the mean ± SEM.

Infiltration of donor T cells to target tissues is indispensable for GVHD. Therefore, we also examined these T cells in the liver and gut, which are GVHD target organs after allo- BMT. We found that donor CD4^+^ and CD8^+^ T cell expansion in liver and gut was similar among these groups at day 21 after allo-BMT (Fig. 6A,B, Fig. 7A,B). Furthermore, no significant differences were found in the proportion of activated T cells in the liver (Fig. 6C,D). In addition, cytokines production by these T cells was not altered (Fig. 6E,H). We also examined effector, memory, naïve T cells, and Tregs, but these subsets were similar among all allogeneic groups (Fig. 6I, Fig. 7C, Supplemental Figs 4–5).

We also examined whether the steroid-refractoriness in our delayed steroid treatment model correlated with persistent T cell expansion, activation and enhanced cytokine production in donor T cells. As in our early steroid treatment models, we did not find any significant alterations of serum cytokine levels, donor T cell expansion, donor T cell subsets, donor T cell activation markers, or cytokine secretion from donor T cells in the spleen, liver or gut when comparing the Ref versus Res groups at day 28 after allo-BMT (data not shown). Collectively, these data suggest that our SR-GVHD models effectively predict survival and therapeutic responses. Unexpectedly, our data also suggest that SR-GVHD may be mediated by a donor T cell independent mechanism.

In this study, we establish steroid refractory murine BMT models using two different clinically relevant BMT models, and two different treatment intervention strategies, early and late DEX treatment. In addition, we used corticosteroid doses that correspond to clinically relevant doses. All of the experiments presented here are required for establishing appropriate models to eventually decipher SR-GVHD pathogenesis and to develop SR-GVHD treatments.

Certainly, our models, like any model system have several limitations including the need to be continually optimized to reflect clinical BMT as clinical practice evolves. Nevertheless, we summarize the main differences between our criteria for murine SR-GVHD and clinical SR-GVHD^9^ in Table 1. First of all, it is difficult to assess organ specific manifestations, such as icterus and the amount of diarrhea in murine GVHD models as is done clinically. Therefore, we used our standard GVHD scoring system which substitutes weight loss, hunching, and activity for gastrointestinal (GI)-GVHD associated symptoms^10^. Therefore, our GVHD scoring system may not be specific nor correlate with histological GVHD grade but this system is used worldwide and consistently reflects murine GVHD severity. Recently, Naserian, *et al*. proposed a new simple, reproducible, and efficient clinical grading system for murine GVHD relying on the binary evaluation of five visual parameters, including diarrhea, and demonstrated correlation with histological grade^11^. Future work will seek to assess if Nasarian’s criteria improve on our model of SR-GVHD.

**Table 1:** Summary of the main differences between our criteria for murine SR-GVHD and clinical SR-GVHD.

	Definition	Differences of our criteria	Similarities of our criteria
Complete response (CR)	Complete resolution of acute GVHD manifestations in all organs.	Clinical score of animal models can’t exactly evaluate organ specific manifestations, such as icterus.	Gaining BW means improvement of manifestations.
Partial response (PR)	Improvement but not complete resolution of acute GVHD manifestations in all initially affected organs without new target organ involvement.	Clinical score of animal models can’t exactly evaluate organ specific manifestations, such as icterus. All other responses between Res and Ref.	Improvement of GVHD clinical score means resolution of acute GVHD manifestation.
Non response (NR)	All other responses or death before response assessment.	Our St groups should be located in NR.	Death before response assessment.

To our surprise, we found that donor T cell characteristics were not significant among steroid responsive, refractory, or stable groups. Future studies will seek to elucidate the critical cellular and molecular mechanisms of glucocorticoid response and resistance. One potential explanation for different responses to DEX is differences in pharmacokinetics between groups, which we will explore in future studies. It would also helpful to assess whether donor derived T cells from newly generated bone marrow or those from peripheral expansion are more affected by glucocorticoid treatment. Another consideration to assess is whether donor T cells exhaustion is different among our groups as there are conflicting reports in this regard^12,13^ following steroid treatment. T cell exhaustion could certainly help explain the clinical differences among our groups. However, why there would be differential exhaustion following steroid treatment in genetically and environmentally homogenous animals is not readily apparent. Nevertheless, our data suggest that immunoregulatory aspects in the context of steroid refractoriness need to be further explored. In addition, these data also suggest that T cell independent mechanisms of tissue tolerance may contribute to SR-GVHD and should be further explored^14,15^.

In conclusion, we established clinically relevant steroid refractory GVHD murine models. Although we have not shown any critical mechanisms, developing a model is the key first steps. We propose using this model to better understand the pathophysiology of SR-GVHD and to develop new therapeutic and prophylactic GVHD strategies.

## Materials and Methods

### Mice

C57BL/6 (B6, H-2^b^, CD45.2^+^), B6 Ly5.2 (H-2^b^, CD45.1^+^), B6D2F1 (H-2^b/d^), and C3H.sw (H-2^b^) mice were purchased from the Jackson Laboratory (Bar Harbor, ME, USA). All animals were cared for according to regulations reviewed and approved by the University Committee on Use and Care of Animals of the University of Michigan, based on University Laboratory Animal Medicine guidelines.

### Bone marrow transplantation (BMT)

BMTs were performed as previously described using well established models^16–18^. Briefly, splenic T cells from donors were enriched with autoMACS (Miltenyi Biotec, Bergisch Gladbach, Germany) utilizing CD90.2 microbeads (Miltenyi Biotec), and bone marrow (BM) was harvested from donor femurs and tibias. B6 and B6D2F1 animals were used as recipients and received 10–11 Gy (^137^Cs source) on day −1 and 3 × 10^6^ CD90.2^+^ T cells along with 5 × 10^6^ BM cells from either syngeneic (B6 or B6D2F1) or allogeneic (C3H.sw or B6) donors on day 0.

### Systemic and histopathological analysis of GVHD

We monitored survival after allo-HCT daily and assessed the degree of clinical GVHD weekly, as described previously^19^. Histopathological analysis of the liver, gastrointestinal (GI) tract, lung, and skin, which are the primary GVHD target organs, was performed as described previously^20^ utilizing a semiquantitative scoring system implemented by a single pathologist (Chen Liu)^20^. After scoring, the codes were broken, and the data compiled.

### Fluorescence-activated cell sorting (FACS) analyses

FACS analyses were performed as previously described^21,22^. Briefly, to analyze donor T cell or myeloid cell expansion and activation markers, splenocytes from transplanted animals were resuspended in FACS wash buffer (2% bovine serum albumin (BSA) in phosphate buffered saline (PBS)) and stained with conjugated monoclonal antibodies (moAbs). The following antibodies were used: FITC-conjugated moAbs to mouse CD45.1 (clone SF1–1.1) and CD229.1 (clone 30C7); PerCP-Cy5.5-conjugated moAbs to mouse CD4 (clone GK1.5), CD8 (clone 53–6.7) and CD11c (clone N418); PE-conjugated moAbs to mouse CD69 (clone H1.2F3), CD62L (clone MEL-14), and CD11b (clone M1/70); and APC-conjugated moAbs to mouse CD44 (clone IM7) and CD25 (clone PC61). All antibodies were purchased from Biolegend (San Diego, CA) except the FITC anti-CD229.1, which was purchased from BD bioscience (San Diego, CA). After staining, the cells were washed twice and fixed with 2% formaldehyde as described previously^21^. Cells were analyzed using a BD Accuri C6 flow cytometer (BD Bioscience). For intracellular cytokine staining, cells were permeabilized after fixation and stained with PE-conjugated IFN-γ (clone XMG1.2, Biolenegd) and granzyme B (clone NGZB, eBioscience), APC-conjugated IL-17A (clone TC11–18H10.1, Biolegend) and perforin (clone eBioOMAK-D, eBioscience) according to manufacturer’s protocol.

### Cytokine enzyme-linked immunosorbent assay (ELISA)

Concentrations of IFN-γ and IL-17A were measured in the serum by ELISA with specific anti-mouse MoAbs for capture and detection utilizing BD OptEIA^TM^ (IFN-γ; BD Biosciences) or ELISA MAX^TM^ (IL-17A; Biolegend). Assays were performed according to each manufacturer’s protocol and read at 450 nm using a microplate reader (Model 3550; Bio-Rad Labs, Hercules, CA). All samples and standards were run in duplicate.

### Statistical analysis

Statistical significance was determined using the Mann-Whitney U test for *in vitro* data, and the Wilcoxon rank test for survival data. A p value <0.05 was considered statistically significant.

## Electronic supplementary material

Supplementary information
