# Supplementary material for: Murine Models of Steroid Refractory Graft-versus-Host Disease
Source: Sci Rep. 2018 Aug 20;8:12475. doi: 10.1038/s41598-018-30814-x (PMC6102256; doi:10.1038/s41598-018-30814-x)

**Original article**

**Murine Models of Steroid Refractory Graft-versus-Host Disease**

Tomomi Toubai^1, 2^*^#†^, Corrine Rossi^1†^, Isao Tawara^3†^, Chen Liu^4^, Cynthia Zajac^1^, Katherine Oravecz-Wilson^1^, Daniel Peltier^5^, Yaping Sun^1^, Hideaki Fujiwara^1^, Julia Wu^1^, Mary Riwes^1^, Israel Henig^1^, Stephanie Kim^1^, Pavan Reddy^1^*

^1^Department of Internal Medicine, Division of Hematology and Oncology, University of Michigan Comprehensive Cancer Center, Ann Arbor, MI, USA.

^2^Department of Internal Medicine III, Division of Hematology and Cell Therapy, Yamagata University Faculty of Medicine, Yamagata, Japan.

^3^Department of Hematology and Oncology, Mie University Graduate School of Medicine, Mie, Japan.

^4^Department of Pathology and Laboratory Medicine, Rutgers-Robert Wood Johnson Medical School, New Brunswick, NJ, USA.

^5^Division of Hematology and Oncology, Department of Pediatrics, University of Michigan, Ann Arbor, MI, USA.

^†^ These authors are equally contributed to this study.

^#^  Current address: Third Department of Internal Medicine, Division of Hematology and Cell Therapy, Yamagata University Faculty of Medicine, Yamagata, Japan.

**Short title**: Mouse model of SR GVHD

**Keywords**: bone marrow transplantation, graft-versus-host disease (GVHD), Steroid refractory GVHD

**Word Count**: Abstract: 161 / Main Text: 1198

**2 Figures and 6 supplemental figures**

***Corresponding Author:**

**Tomomi Toubai**

Department of Internal Medicine III, Division of Hematology and Cell Therapy, Yamagata University Faculty of Medicine, 2-2-2 Iida-Nishi, Yamagata, 990-9585, Japan.

E-mail: [toubai@med.id.yamagata-u.ac.jp](mailto:toubai@med.id.yamagata-u.ac.jp)

Tel: +81-23-628-5316, Fax: +81-23-628-5318

**Supplemental Figure 1: Early SR-GVHD model.**

(a-b) B6 WT animals received 10 Gy on day -1 and were transplanted with 3.0x10^6^ CD90.2^+^ splenic T cells along with 5x10^6^ TCD-BM from either syngeneic B6 or allogeneic MHC matched multiple miHAs mismatched C3H.sw donors. Recipient animals were treated intraperitoneally with 0.1mg/kg/day of dexamethasone (DEX) from day +7 thru +21 after allo-BMT. The allogeneic DEX treated animals shown in Figure 1B were divided into three groups based on our criteria, such as steroid responsive (Res), stable (St) or steroid refractory (Ref) GVHD. GVHD clinical score (a) and percent BW loss (b) in figure 2B. n=3-14 per group. Data shown are pooled from four independent experiments. Error bars show the mean ± SEM.

**Supplemental Figure 2: Application of steroid responsiveness criteria to allogeneic untreated animals.**

(a-c) B6 WT animals received 10 Gy on day -1 and were transplanted with 3.0x10^6^ CD90.2^+^ splenic T cells along with 5x10^6^ TCD-BM from either syngeneic B6 or allogeneic MHC matched multiple miHAs mismatched C3H.sw donors. The allogeneic untreated control animals shown in Figure 1B were divided based on our criteria, such as steroid responsive (Res), stable (St) or steroid refractory (Ref) GVHD. Survival (a), GVHD clinical score (b), and the percentage of BW loss (c) are shown. *p<0.05 when the animals with Ref criteria were compared with St one. n=5-10 per group. Data shown are pooled from four independent experiments. Error bars show the mean ± SEM.

**Supplemental Figure 3: Splenic donor T cell subpopulation in DEX treated animals.**

(a-f) B6 WT animals received 10 Gy on day -1 and were transplanted with 3.0x10^6^ CD90.2^+^ splenic T cells along with 5x10^6^ TCD-BM from either syngeneic B6 or allogeneic MHC matched multiple miHAs mismatched C3H.sw donors. Recipient animals were treated intraperitoneally with 0.1mg/kg/day of dexamethasone (DEX) from day +7 thru +21 after allo-BMT. The percentage of effector T cells (CD44^+^CD62L^-^)(a-b), central memory T cells (CD44^+^CD62L^+^)(c-d), and naïve T cells (CD44^-^CD62L^+^)(e-f) in donor CD229.1^+^CD4^+^ (a, c, e) or CD8^+^ T cells (b, d, f) in the spleen on day 14 of DEX (day 21 after allo-BMT) from the different groups presented in Figure 2B is shown. n=3-10 per group. Data are pooled from five experiments. ***p<0.001, **p<0.01, *p<0.05. Error bars show the mean ± SEM.

**Supplemental Figure 4: Liver donor T cell subpopulation in DEX treated animals.**

(a-f) B6 WT animals received 10 Gy on day -1 and were transplanted with 3.0x10^6^ CD90.2^+^ splenic T cells along with 5x10^6^ TCD-BM from either syngeneic B6 or allogeneic MHC matched multiple miHAs mismatched C3H.sw donors. Recipient animals were treated intraperitoneally with 0.1mg/kg/day of dexamethasone (DEX) from day +7 thru +21 after allo-BMT. The percentage of effector T cells (CD44^+^CD62L^-^)(a-b), central memory T cells (CD44^+^CD62L^+^)(c-d), and naïve T cells (CD44^-^CD62L^+^)(e-f) in donor CD229.1^+^CD4^+^ (a, c, e) or CD8^+^ T cells (b, d, f) in the liver on day 14 of DEX (day 21 after allo-BMT) from the different groups in Figure 2B is shown. n=3-11 per group. Data are pooled from five experiments. ***p<0.001, *p<0.05. Error bars show the mean ± SEM.

**Supplemental Figure 5: Intestinal donor T cells in DEX treated animals.**

(a-f) B6 WT animals received 10 Gy on day -1 and were transplanted with 3.0x10^6^ CD90.2^+^ splenic T cells along with 5x10^6^ TCD-BM from either syngeneic B6 or allogeneic MHC matched multiple miHAs mismatched C3H.sw donors. Recipient animals were treated intraperitoneally with 0.1mg/kg/day of dexamethasone (DEX) from day +7 thru +21 after allo-BMT. The percentage of effector T cells (CD44^+^CD62L^-^)(a-b), central memory T cells (CD44^+^CD62L^+^)(c-d), and naïve T cells (CD44^-^CD62L^+^)(e-f) in donor CD229.1^+^CD4^+^ (a, c, e) or CD8^+^ T cells (b, d, f) in the intestine on day 14 of DEX (day 21 after allo-BMT) from the different groups from Figure 2B is shown. n=3-11 per group. Data are pooled from five experiments. ***p<0.001, *p<0.05. Error bars show the mean ± SEM.


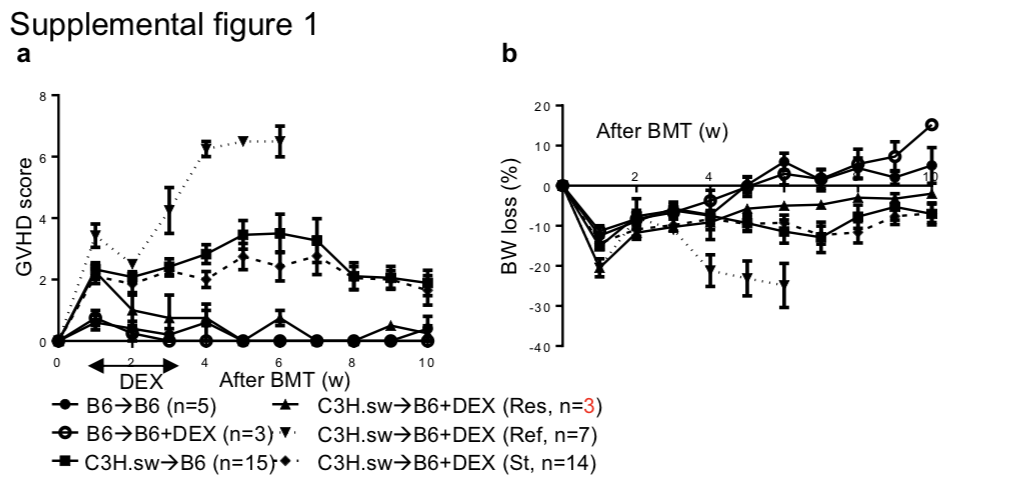


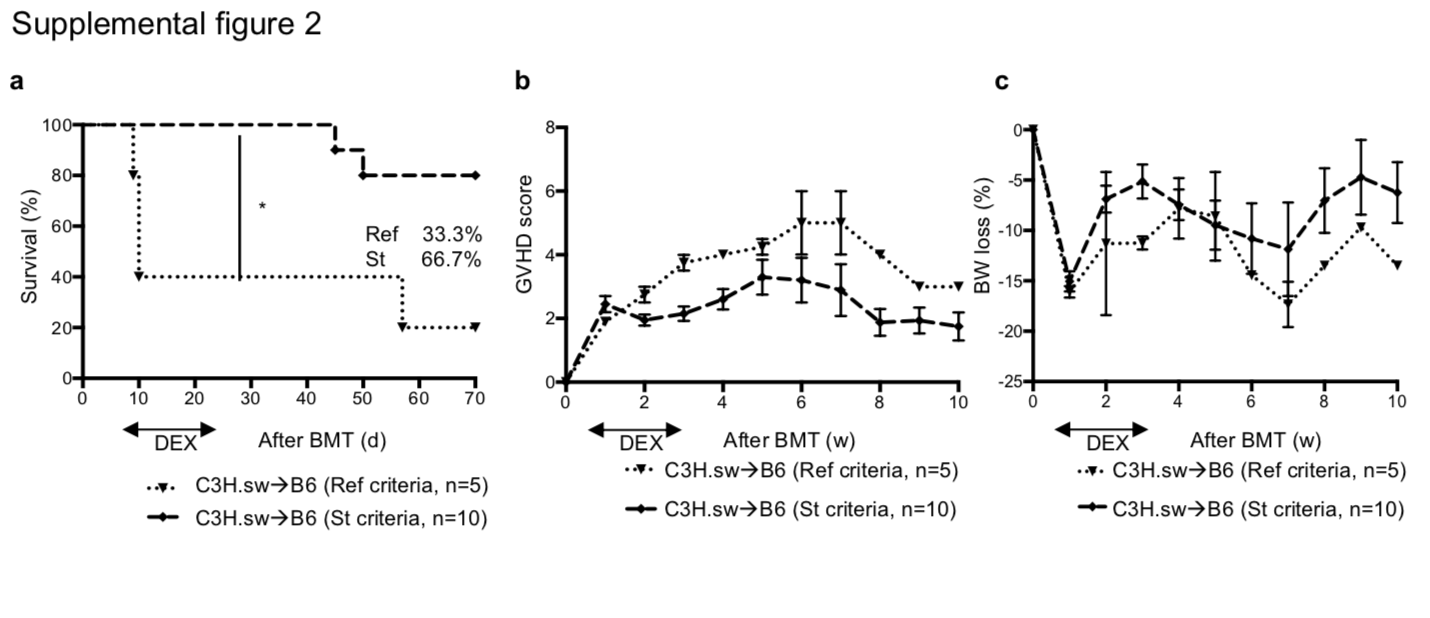


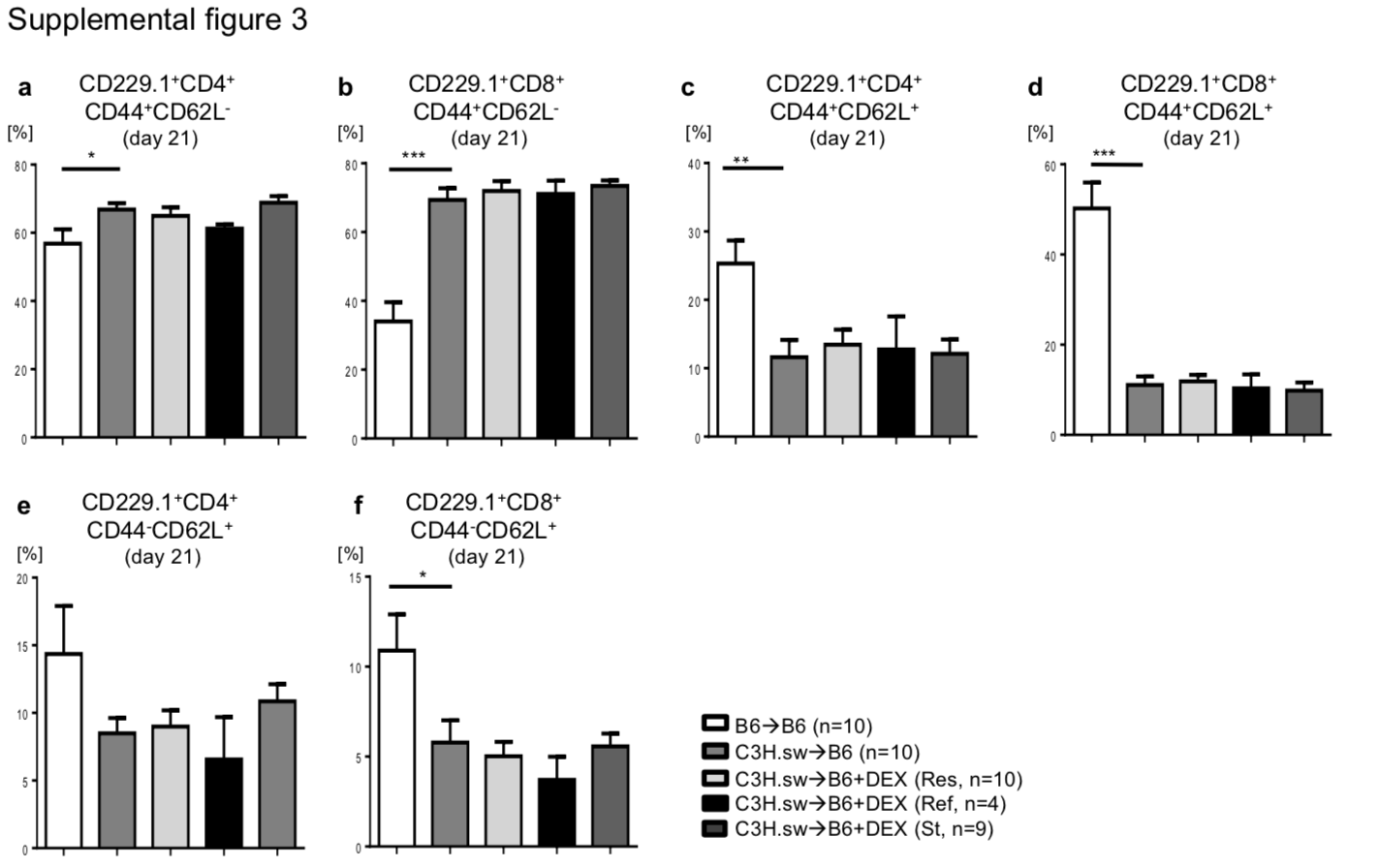


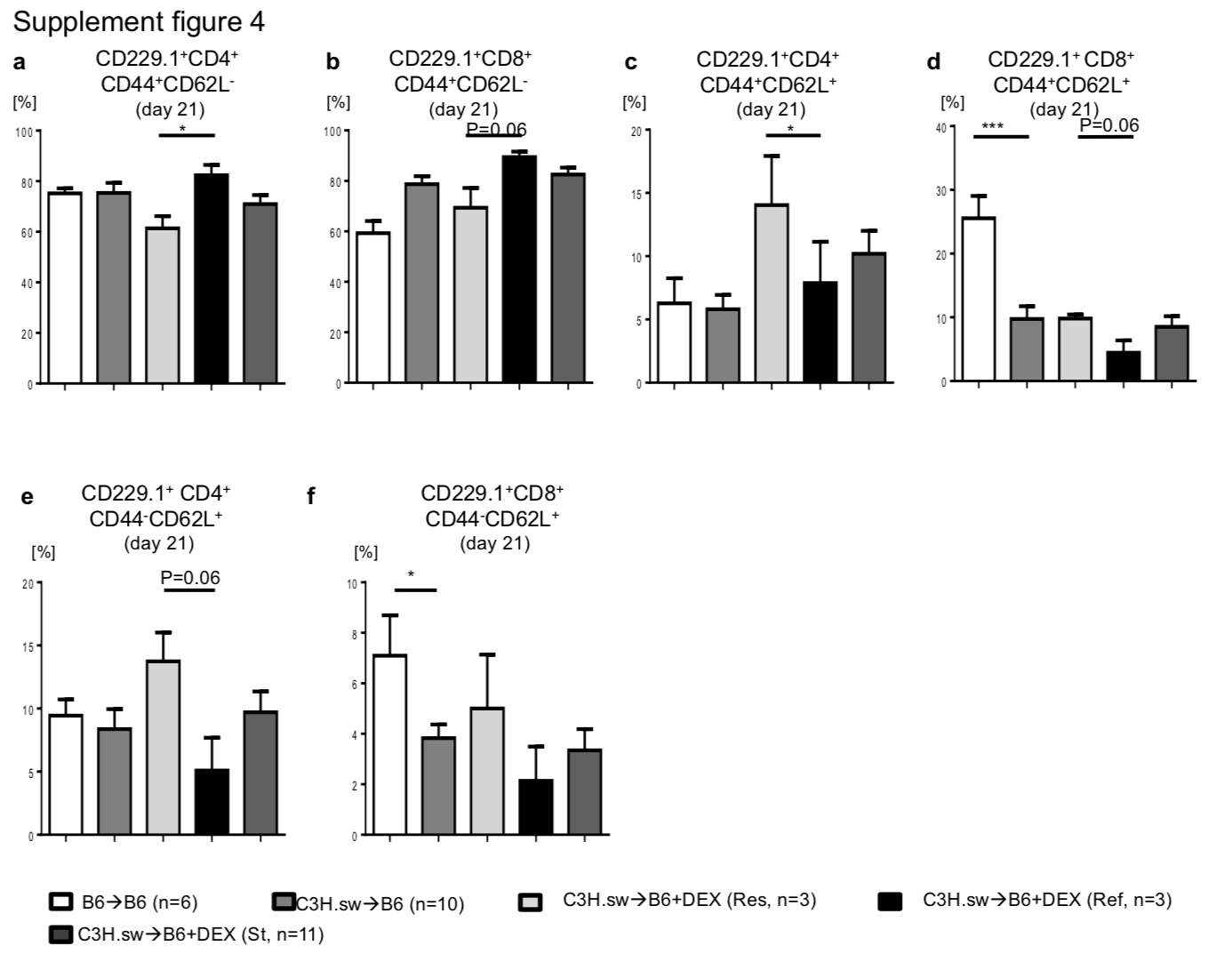


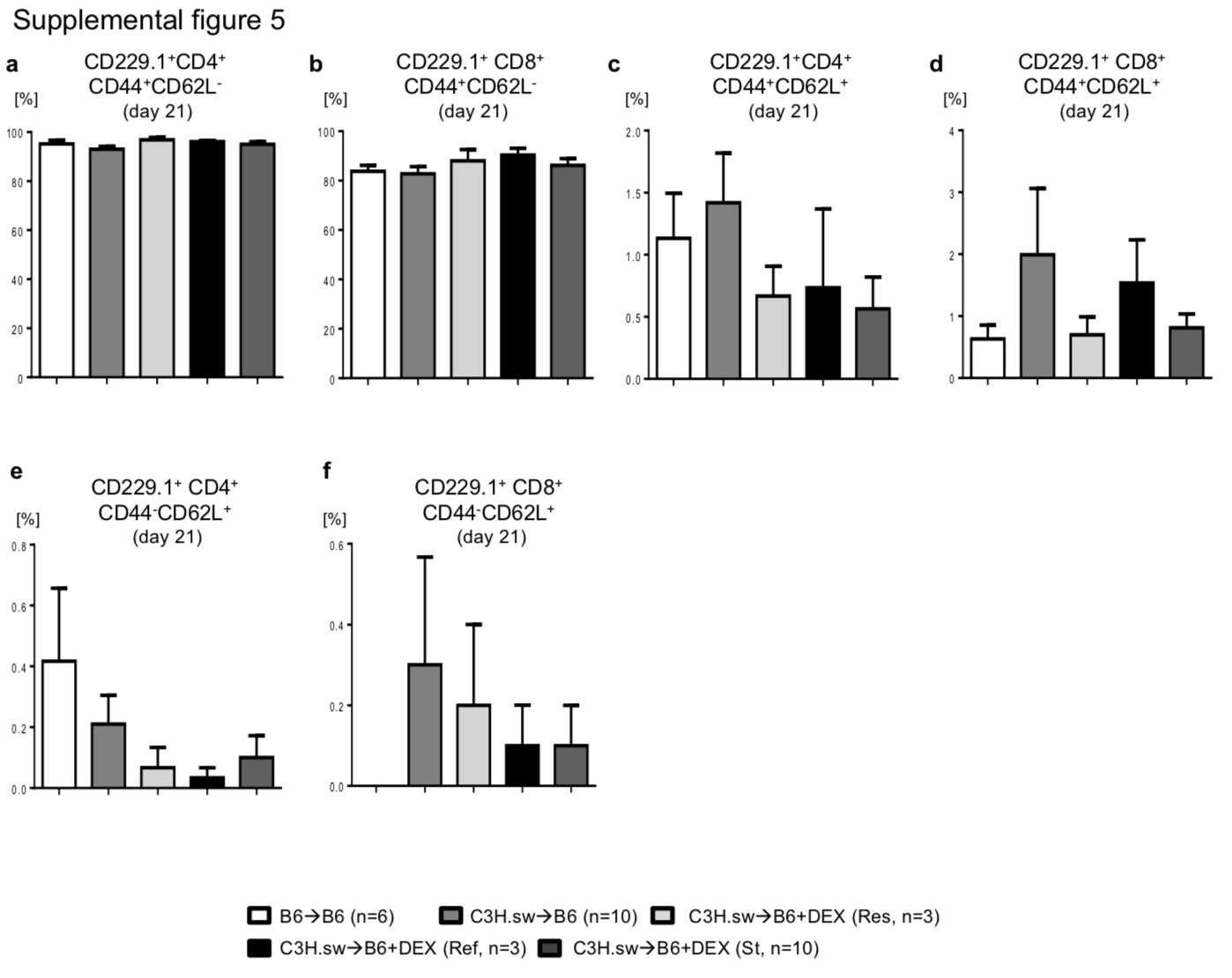

Supplement: Supplementary file 1 — Supplementary information [file 41598_2018_30814_MOESM1_ESM.docx]
